# The Possible Roles of Biological Bone Constructed with Peripheral Blood Derived EPCs and BMSCs in Osteogenesis and Angiogenesis

**DOI:** 10.1155/2016/8168943

**Published:** 2016-04-18

**Authors:** Li Wu, Xian Zhao, Bo He, Jie Jiang, Xiao-Jie Xie, Liu Liu

**Affiliations:** ^1^Medical Imaging Department, The First Affiliated Hospital, Kunming Medical University, Kunming 650032, China; ^2^Plastic Surgery Department, The First Affiliated Hospital, Kunming Medical University, Kunming 650032, China

## Abstract

This study aimed to determine the possible potential of partially deproteinized biologic bone (PDPBB) seeded with bone marrow stromal cells (BMSCs) and endothelial progenitor cells (EPCs) in osteogenesis and angiogenesis. BMSCs and EPCs were isolated, identified, and cocultured* in vitro*, followed by seeding on the PDPBB. Expression of osteogenesis and vascularization markers was quantified by immunofluorescence (IF) staining, immunohistochemistry (IHC), and quantitive real-time polymerase chain reaction (qRT-PCR). Scanning electron microscope (SEM) was also employed to further evaluate the morphologic alterations of cocultured cells in the biologic bone. Results demonstrated that the coculture system combined with BMSCs and EPCs had significant advantages of (i) upregulating the mRNA expression of VEGF, Osteonectin, Osteopontin, and Collagen Type I and (ii) increasing ALP and OC staining compared to the BMSCs or EPCs only group. Moreover, IHC staining for CD105, CD34, and ZO-1 increased significantly in the implanted PDPBB seeded with coculture system, compared to that of BMSCs or EPCs only, respectively. Summarily, the present data provided evidence that PDPBB seeded with cocultured system possessed favorable cytocompatibility, provided suitable circumstances for different cell growth, and had the potential to provide reconstruction for cases with bone defection by promoting osteogenesis and angiogenesis.

## 1. Introduction

In china, there are over three million patients with the bone defects of cranium, mandibula, and limbs. This number is increasing 10% each year with population aging. Taking advantage of modern biological materials, current grafting treatments are extensively applied to clinic cases. However, the specific shortcomings and limitations of those grafting materials hold back the clinic research and application of engineering bone [1, 2].

Reports reveal that crucial steps contributing to engineering bone development or restructure include the appropriate composite biomaterials for bone regeneration [3, 4], as well as prepared reparative cells [5, 6]. By far multiple studies have confirmed the great potential of bone marrow stromal cells (BMSCs) in promoting regeneration of bone defects both in animal models and in humans [7, 8]. Additionally, evidences support that endothelial progenitor cells (EPCs) contribute to revascularization* in vitro* and* in vivo* and enhance bone formation to bridge bone defects for bone repair [9, 10]. Biomaterial scaffold, which is necessary for reparative cells, has the ability to influence cell adhesion and biological behavior (morphology, proliferation, and differentiation of neighbouring cells) [11].

One of the most important criteria for an ideal bone tissue engineering scaffold is that the scaffold consists of a highly interconnected porous network with pore sizes large enough for cell migration, fluid exchange, and eventually tissue ingrowth and vascularization [12]. Although synthetic calcium phosphate ceramics with their excellent biocompatibility are designed to mimic the native extracellular matrix as closely as possible [13], the porous network of the artificial bone scaffold still cannot compare to natural bone in terms of structure and function. There is a growing need to provide alternatives to traditional bone grafting. Deproteinized bovine bone produced from natural materials, known as inorganic porous bone, has gained wide acceptance for various medical applications for autologous bone grafts [14]. Even so, deproteinized bovine bone has no capability of enhancing bone regeneration because it is not osteoinductive. Composite biomaterial combined with both of the reparative cells and the biological bone is a promising strategy for bone regeneration. However, little is known about the effect and influence of composite materials containing a partially deproteinized biologic bone (PDPBB) fraction on cells with osteogenesis capabilities.

In the present study, we prepared biological engineering bone PDPBB seeded with cocultured BMSCs and EPCs and then determined its potential for osteogenesis and angiogenesis. Peripheral blood derived EPCs and BMSCs were isolated, identified, and seeded onto the PDPBB. The efficiency of BMSCs, EPCs alone, and the coculture system on the osteogenesis and angiogenesis was dynamically monitored both* in vitro* and* in vivo* following transplantation. Briefly, the third-generation EPCs and BMSCs were cocultured and prepared for alkaline phosphatase (ALP) and osteocalcin (OC) quantitive detection by IHC, after 3, 7, and 14 days of culture. The mRNA levels of Osteonectin, Osteopontin, vascular endothelial cell growth factor (VEGF), and Collagen Type I were also evaluated by using qRT-PCR. PDPBB was prepared from fresh porcine spine bone modified with fibronectin and then seeded with EPCs, BMSCs alone, and coculture system, respectively. The different engineering bones were then implanted into the muscle of rabbits. The osteogenesis and angiogenesis were evaluated by IHC staining with CD34, CD105, and ZO-1 after 14, 28, and 60 days of implantation. SEM was also employed for morphological detection of PDPBB seeded with different cells.

## 2. Materials and Methods

### 2.1. Animal and Ethics Statement

Twelve New Zealand male rabbits with 2.5 kg mean weight were employed in the present research. Animal use and care were in accordance with the animal care guidelines, which conformed to the Guide for the Care and Use of Laboratory Animals published by the US National Institutes of Health (NIH, publication number 85-23, revised 1996) and the Care and Use Guidelines of Experimental Animals established by the Ministry of Medicine of Yunnan, China. The ethics committee of Kunming Medical University specifically approved this study (permit number: km-edw-2014708). All surgical procedures were performed under chloral hydrate anesthetised, and all efforts were made to minimize suffering.

### 2.2. Cell Isolate and Characterization

Adult New Zealand rabbits were anesthetised by intraperitoneal injection with chloral hydrate (2 mL/kg). EPCs were isolated from rabbit peripheral blood and cultured in endothelial cell medium (ECM) [15]. After six days' culture, EPCs were identified by immunofluorescence staining for CD34 (hematopoietic progenitor antigen) (1 : 1000, Abcam, CA, UK), CD133 (PROM1) (1 : 500, Santa Cruz, USA), and vWF (von Willebrand Factor) (1 : 800, Santa Cruz, USA). Bone marrow stromal cells (BMSCs) were isolated from the same rabbit bone marrow blood and cultured in DMEM containing 15% fetal bovine serum [16]. Cells of the third passage were harvested for further use.

The immunofluorescence staining for CD34 (1 : 500), CD29 (integrin beta-1) (1 : 400, Santa Cruz, USA), and CD90 (Thy-1) (1 : 200, Vector, USA) was also used for EPCs identification.

The coculture system was reconstructed as described as follows. EPCs and BMSCs were cocultured with the ratio 1 : 2 in diet culture medium including 10% FBS, by gently pipetting up and down, and seeded on six-well plate at a density of 2 × 10^5^ cells/mL. The medium was replaced once per three days. The pure EPCs or BMSCs culture served as control.

### 2.3. Immunofluorescence (IF) Staining

The IF staining was performed as described before [17]. Anti-rabbit CD34 (1 : 500), CD133 (1 : 500), vWF (1 : 200), CD29 (1 : 800), and CD90 (1 : 200) were used. Cells were then stained with a secondary antibody for 2 h. Goat anti-rabbit IgG and Alexa Fluor® 488 Donkey Anti-Rabbit IgG (Life Technologies) were used for second antibody staining and then counterstained with the nuclear dye 4′,6-diamidino-2-phenylindole (DAPI) (Biotium, Hayward, CA, USA). All images were taken with a Leica fluorescence microscope.

### 2.4. Coculture System of BMSCs and EPCs* In Vitro*

The coculture system combined with EPCs and BMSCs referred to others described before [18]. Briefly, to determine the optimal ratio of EPCs and BMSCs for cocultured system, seven groups were designed, including BMSCs alone, EPCs alone, and EPCs : BMSCs at ratios of 2 : 1 (6 × 10^4^ : 3 × 10^4^ cells), 1 : 2 (3 × 10^4^ : 6 × 10^4^ cells), and 1 : 1 (5 × 10^4^ : 5 × 10^4^ cells). EGM (EGM-2 medium supplemented with growth factor bullet kit (Lonza, Cologne, Germany))/complete medium (CM), which is DMEM (Gibco), supplemented with 10% fetal bovine serum (FBS, Gibco), L-glutamine (2 mmol/L), and penicillin (100 U/mL) and EGM/osteogenic (OS) media were employed. Cells were plated at 1 × 10^5^ cells per well in 12-well plates and induced with EGM/complete media. Cocultured group combined with EPCs : BMSCs at ratios of 1 : 2 achieved the most excellent proliferation activity among the groups and was significantly different compared to others (see Figure S1 at Supplementary Material available online at http://dx.doi.org/10.1155/2016/8168943).

### 2.5. qRT-PCR

On days 3, 7, and 14 of culture, a total of 1 × 10^6^ cells from pure EPCs, BMSCs, and coculture system were obtained (*n* = 7). The mRNA expressions of multiple genes in cultured cells, including VEGF, Osteopontin, Osteonectin, and Collagen Type I, were detected by qRT-PCR. All experiments were performed in triplicate. The protocol was according to XiYang et al. [19]. Briefly, Trizol reagent (Invitrogen, USA) was used to isolate total RNA of sample. cDNA was synthesized by using Oligo(dT)^18^ and MMLV reverse transcriptase (Promega, Madison, WI). Gene primers were synthesized by Shengon Ltd. Company (Shanghai, China). The primers were recorded in Table 1. qRT-PCR protocol was applied using ABI 5700 instrument (Bio-Rad Laboratories, Shanghai, China). The melting curve analysis was performed to confirm the specificity of the amplification products. PCR was performed by the denaturation step at 95°C for 3 minutes, followed by 35 cycles of 95°C for 15 seconds, annealing for 15 seconds, and 72°C for 30 seconds. Fluorescent signals obtained from PCR products were recorded at 85.5°C for 5 seconds. Relative CT method was employed to compare difference between samples. The fold decrease/increase was determined relative to a blank control after normalizing to a housekeeping gene using 2^−ΔΔCT^ [20].

### 2.6. Osteoblast Identification by ALP Staining and OC Assay

Osteoblasts were identified by using an ALP (Jackson, USA) or OC (Jackson, USA) staining kit according to the manufacturer's instructions, and osteogenic differentiation of EPCs, BMSCs, and coculture system was confirmed. The stained cells were then photographed with a camera [21].

The levels of ALP and OC were determined according to the manufacturer's instructions (Jackson, USA). In order to normalize these markers expressions for quantification, medium supernatant from each subgroup was collected to evaluate the ALP and OC levels at the same density of 1 × 10^7^ cells per well. After 3, 7, or 14 days of culture, the medium supernatant from each group was collected and used to evaluate the ALP and OC level [21]. The OD was read at 450 nm using ELISA plate reader (Bio-Rad). All samples were assayed in duplicate.

### 2.7. Reconstruction of Biologic Bone Seeded with Cells

The PDPBB treated with fibronectin [22, 23] was prepared from fresh porcine spine bone modified with fibronectin as described in Table 2. A total of 168 pieces of PDPBB, with size of 0.5 × 0.5 × 0.1 cm each, were prewetted with medium solutions for 30 min.

Rabbit pure EPCs (0.5 × 10^6^/mL 30 *μ*L EPCs), BMSCs (0.5 × 10^6^/mL 30 *μ*L BMSCs), and coculture system (20 *μ*L BMSCs + 10 *μ*L EPCs) were seeded into PDPBB to reconstruct tissue-engineered bone* in vitro*, respectively [20, 24]. The optimal cells seeding density described above achieved an excellent adhesion and proliferation activity on PDPBB [24].

The proliferation of EPCs, BMSCs, or cocultured system seeded on PDPBB was also determined by Wst-1 assay. Coculture seeded group achieved the most excellent proliferation activity among the groups and was significantly different compared to others (Supplemental Data, Figure S2).

### 2.8. PDPBB Transplantation

The twelve rabbits above from which we extracted peripheral blood and bone marrow for cell culture underwent the surgery of PDPBB transplanted back into their body. The engineering bone groups, including PDPBB + EPCs, PDPBB + BMSCs, and PDPBB + coculture system, were, respectively, transplanted into the muscle of the upper limbs of animal. Briefly, the rabbits were anaesthetized with intraperitoneal injection of 3.6% chloral hydrate (2 mL/kg). The skin and subcutaneous tissue of both upper limbs were incised, and then the sarcolemma and muscle were bluntly dissected by Wallerian clamp. Muscle bag with 1 × 1 × 1 cm volume was enlarged by forceps for engineered bone transplantation. After operation, the rabbits received daily injections of amoxicillin (dosage at 20 mg) for 3 days.

### 2.9. Immunohistochemistry (IHC)

At 14, 28, and 60 days after transplantation, the rabbits in engineering bone groups were under anesthesia and the engineering bones were isolated for IHC analysis. After anesthesia with 3.6% chloral hydrate, transplanted engineering bones were carefully isolated and fixed in 4% paraformaldehyde solution. They were then embedded with resin. Sections of 4 *μ*m thickness were cut in a hard tissue microtome, collected and flatted in water bath at 60°C, dredged on slide, and baked in oven at 50°C. The following protocol was described as others [25]. After immersing in 0.01 M PBS (containing 5% goat serum and 0.3% TritonX-100 solution) at 37°C for 30 min, they were subsequently incubated at 4°C overnight with 2% goat serum containing goat polyclonal antibodies CD34 (1 : 800), CD105 (endoglin) (1 : 1000, Santa Cruz), or ZO-1 (Zonula occludens-1) (1 : 500, Santa Cruz), respectively. Immunoreactive products were observed and photographed with a light microscope coupled with a computer assisted video camera (Leica DMIRB, Germany).

### 2.10. Scanning Electron Microscope (SEM)

Field Emission SEM S-4800 (Hitachi, Japan) was employed to characterize the PDPBB, as well as the morphology and behavior of the cells grown in the PDPBB. Prior to imaging, the cells were fixed with 2.5% glutaraldehyde and samples were dehydrated in a graded ethanol series and sputter-coated with gold for 15–20 s. All samples were analyzed at 15 kV [26].

### 2.11. Statistical Analysis

SPSS v14.5 (SPSS Inc., Chicago, IL) was used for statistical analysis. Data are presented as means ± standard deviation (SD). Two-way ANOVA were used to determine statistically significant differences between groups, followed by Bonferroni posttests. A *P* value of less than 0.05 was considered statistically significant (*P* < 0.05).

## 3. Result

### 3.1. Isolation and Characterization of MSCs and EPCs

**Figure 1:**
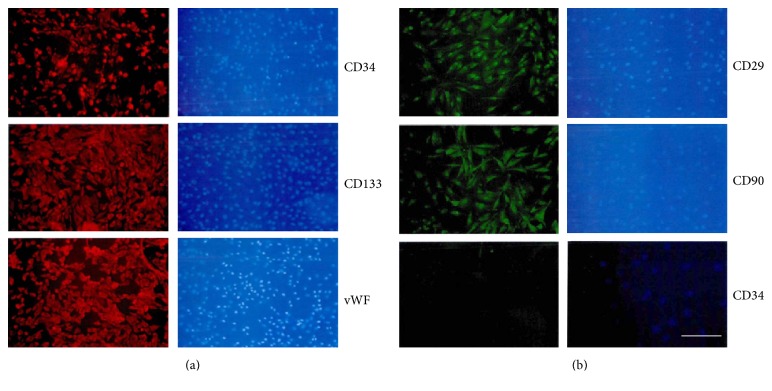
Characterization of EPCs and BMSCs by immunofluorescence staining. (a) IF analysis for EPCs cell surface markers, CD34, CD133, and vWF (red); nucleuses were stained by DAPI (blue), Magnifications: 100x, scale bar: 100 *μ*m; (b) IF analysis for BMSCs cell surface markers, CD29, CD90 (red, Magnifications: 100x, and scale bar: 100 *μ*m), and CD34 (green); nucleuses were stained by DAPI (blue) (Magnifications: 400x, scale bar: 25 *μ*m).

The cells were analyzed and identified by IF staining for EPCs and BMSCs cell surface markers at passage 3. For EPCs, we analyzed the early hematopoietic progenitor cell marker by IF. Results showed that EPCs expressed a cell surface protein profile positive for CD34, CD133, and vWF (Figure 1(a) and Supplemental Data, Figure S3). As shown in Figure 1(b), BMSCs expressed a cell surface protein profile positive for CD29 and CD90 and negative for CD34. IF staining showed that EPCs were CD34+/CD133+/vWF+, while BMSCs were CD29+/CD90+/CD34−.

### 3.2. Effect of Cocultured System on ALP Activity

**Figure 2:**
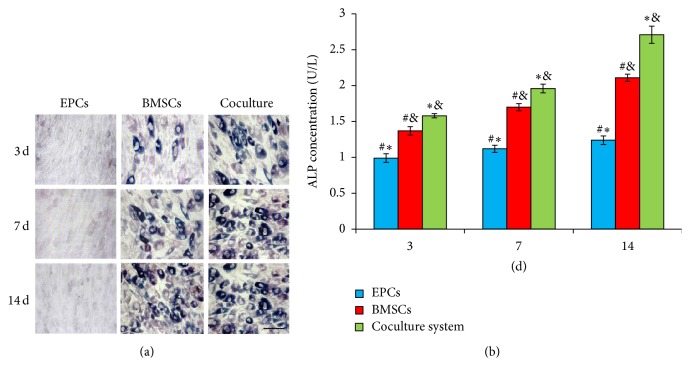
ALP analysis in three groups after 3, 7, and 14 days of cell culture. (a) ALP staining in EPCs, BMSCs, and cocultured groups, Magnifications: 400x, scale bar: 25 *μ*m. (b) ALP content evaluated by quantitive analysis. Values plotted are means ± SD (*n* = 6). ^*∗*^ *P* < 0.05, versus BMSCs group; ^&^ *P* < 0.05, versus EPCs group; ^#^ *P* < 0.05, versus coculture group.

At 3 days, ALP stain was present negative either in EPCs group or in BMSCs group, while it showed partially positive staining in cocultured group. At 7 days, ALP stain of the EPCs group still appeared negative. However, positive cells were present in either BMSCs or coculture group. Moreover, there were more positive cells observed in the coculture group compared to that of BMSCs group (Figure 2(a) and Supplemental Data, Figure S4). At 14 days, deep ALP stain was observed in confluent cells in coculture group, while light stained cells of ALP were scattered in BMSCs only group. Till 14 days, there were no ALP positive cells observed in EPCs only group (Figure 2(a)).

Quantitive analysis in ALP content by CurveExpert 1.4 software was performed. The results showed that ALP content of BMSCs and coculture group increased gradually from 3 to 14 days after culture, and the level peaked in coculture group at 14 days, when compared to EPCs or BMSCs only group, respectively (*P* < 0.05, Figure 2(b)). In EPCs group, the ALP level revealed no significant difference between each time point (*P* > 0.05). Through the whole experimental period, content of ALP detected in coculture group was more compared to BMSCs only group (*P* < 0.05, Figure 2(b)).

### 3.3. Effect of Cocultured System on OC Expression

**Figure 3:**
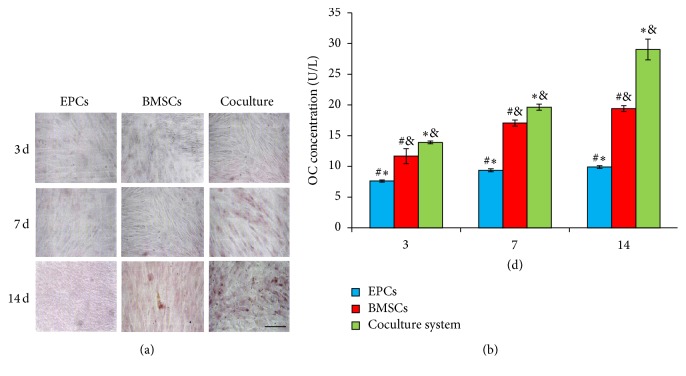
OC analysis in three groups after 3, 7, and 14 days of cell culture. (a) OC staining in EPCs, BMSCs, and cocultured groups, Magnifications: 200x, scale bar: 50 *μ*m. (b) OC content evaluated by quantitive analysis. Values plotted are means ± SD (*n* = 6). ^*∗*^ *P* < 0.05, versus BMSCs group; ^&^ *P* < 0.05, versus EPCs group; ^#^ *P* < 0.05, versus coculture group.

At 3 days, no OC stain was detected in three groups. At 7 days, there were partially positive staining in cocultured group and light pink in BMSCs group. OC stain of the EPCs group still appeared negative. At 14 days, confluent OC positive cells were observed in coculture group, while light stained cells of OC were scattered in BMSCs only group. Moreover, few calcium nodes were also observed in the coculture group but not in BMSCs alone one. There were no positive OC staining cells observed in EPCs only group, until 14 days (Figure 3(a)).

The OC content of each group increased gradually from 7 to 14 days, and the level of OC was the highest in coculture group on each time point, compared to EPCs or BMSCs only group, respectively (*P* < 0.05, Figure 3(b)). There are statistically significant differences between each group.

### 3.4. Alteration in mRNA Expression of Osteoblast and Angiogenesis Genes

**Figure 4:**
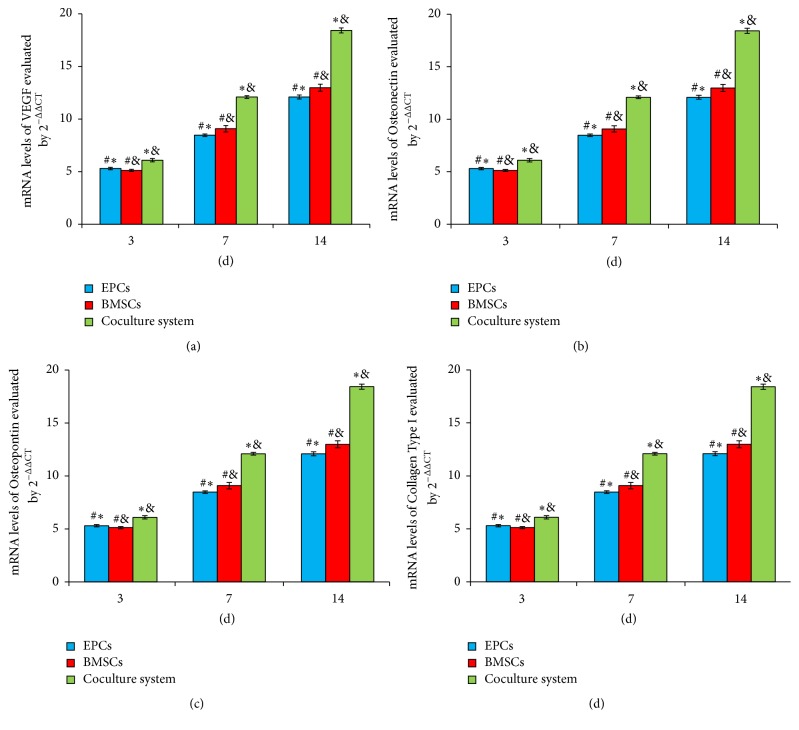
mRNA expression of genes associated with osteoblast and angiogenesis. Relative mRNA levels of VEGF (a), Osteonectin (b), Osteopontin (c), and Collagen Type I (d) evaluated by qRT-PCR in three different groups. Values plotted are means ± SD (*n* = 7). ^*∗*^ *P* < 0.05, versus BMSCs group; ^&^ *P* < 0.05, versus EPCs group; ^#^ *P* < 0.05, versus coculture group.

The mRNA levels of VEGF, Osteonectin, Osteopontin, and Collagen Type I, detected by qRT-PCR, were gradually increased from 3 to 7 to 14 days. Compared to BMSCs or EPCs alone group, there were increased VEGF, Osteonectin, Osteopontin, and Collagen Type I mRNA levels detected in cocultured group (*P* < 0.05, Figure 4).

As shown in Figure 4, compared to BMSCs group, the level of VEGF gene was upregulated significantly in cocultured or EPCs group (*P* < 0.05). However, there was no significant difference between cocultured and EPCs group at 3 days (*P* > 0.05). From 7 to 14 days, the cocultured system had significant advantages of upregulating the mRNA expression of VEGF compared to the EPCs alone group (*P* < 0.05, Figure 4(a)).

At 3 days, the mRNA expression of Collagen Type I was upregulated significantly in cocultured or BMSCs group (*P* < 0.05). From 7 to 14 days, the cocultured system had significant advantages of upregulating the mRNA expression of Collagen Type I compared to the BMSCs or EPCs alone group (*P* < 0.05). There was no significant difference between BMSCs and EPCs group (*P* > 0.05, Figure 4(d)). A similar tendency was observed in the expression of Osteopontin and Osteonectin genes in three groups (Figures 4(b) and 4(c)).

### 3.5. The Morphology of Cells on Heterotopia Bone Grafting

**Figure 5:**
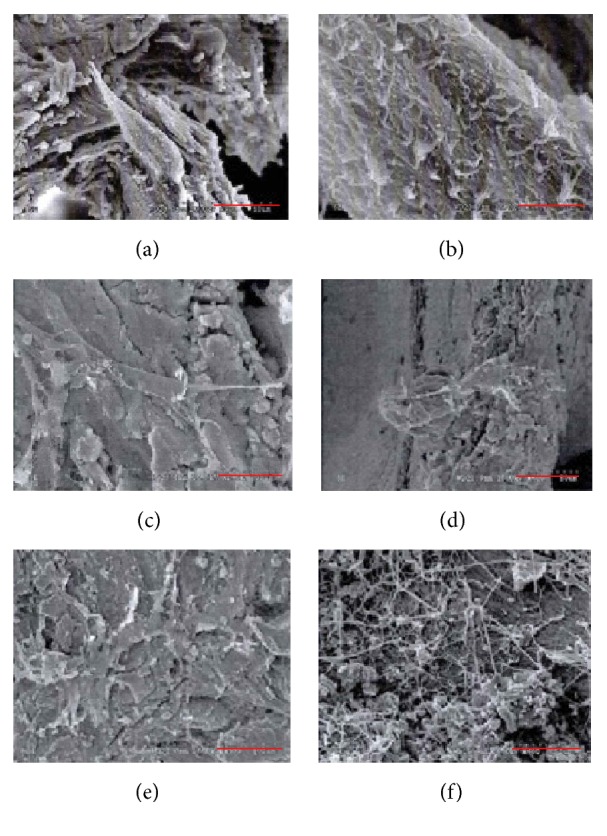
Representative SEM micrographs of PDPBB seeded with different cells. The micrographs show the proliferation and spreading of cocultured EPCs and BMSCs ((e) and (f)) after seeding in PDPBB scaffolds. ((a) and (b)) PDPBB with EPCs alone; ((c) and (d)) PDPBB with BMSCs alone; ((e) and (f)) PDPBB with cocultured EPCs and BMSCs. Scale bar: (a), (b), (e), and (f): 15 *μ*m and (c) and (d): 5 *μ*m.

In PDPBB seeded with EPCs, few polygonal endotheliocytes attached to the surface of PDPBB under SEM. It could be observed that flat cells with irregular shape attached to the surface of PDPBB + BMSCs by pseudopodia. The BMSCs were scattered and spread on the PDPBB surface. Of note, compared to other PDPBB + EPCs or PDPBB + BMSCs only, a large number of cocultured cells appeared better adhered and significantly grown in number to link as flakiness on the surface of the PDPBB + cocultured system (Figure 5).

### 3.6. Effect of Cocultured EPCs and BMSCs on Microvascular Angiogenesis

**Figure 6:**
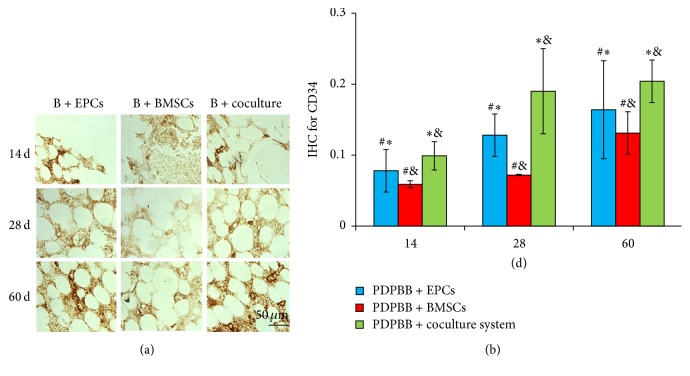
IHC detection and quantitive analysis of CD34 in PDPBB groups after implantation. (a) CD34 staining in PDPBB seeded with EPCs, BMSCs, and cocultured groups, respectively; B + EPCs, PDPBB + EPCs; B + BMSCs, PDPBB + BMSCs; B + coculture, PDPBB + cocultured system. (b) Quantitive analysis of CD34 staining in three groups after implantation. Values plotted are means ± SD (*n* = 3). ^*∗*^ *P* < 0.05, versus PDPBB + BMSCs group; ^&^ *P* < 0.05, versus PDPBB + EPCs group; ^#^ *P* < 0.05, versus PDPBB + coculture group.

The microvascular vascularization after biological bone transplantation* in vivo* was observed by IHC staining of CD34, CD105, and ZO-1, respectively. At 14 days after transplantation, granulation tissues grew into the cavity of engineering bone grafting. There were some positive cells and microvascular structures observed in group EPCs and fewer positive cells were observed in BMSCs group and microvascular structures were observed in this group. The expressions of CD34, CD105, and ZO-1 kept the highest in cocultured group, which showed significant difference compared with the control groups (*P* < 0.05, Figure 6).

**Figure 7:**
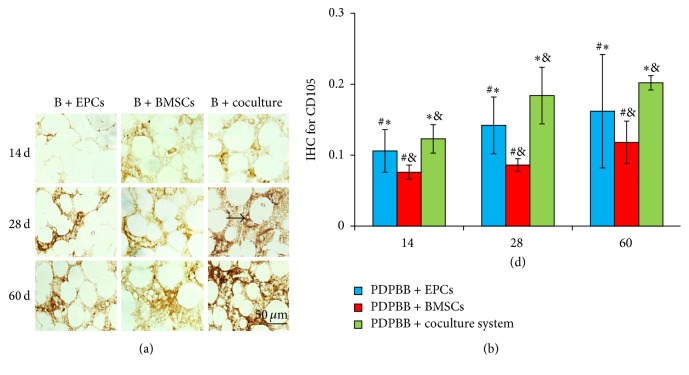
IHC detection and quantitive analysis of CD105 in PDPBB groups after implantation. (a) CD105 staining in PDPBB seeded with EPCs, BMSCs, and cocultured groups, respectively; B + EPCs, PDPBB + EPCs; B + BMSCs, PDPBB + BMSCs; B + coculture, PDPBB + cocultured system. (b) Quantitive analysis of CD105 staining in three groups after implantation. Arrow showed the representative IHC staining of CD105. Values plotted are means ± SD (*n* = 3). ^*∗*^ *P* < 0.05, versus PDPBB + BMSCs group; ^&^ *P* < 0.05, versus PDPBB + EPCs group; ^#^ *P* < 0.05, versus PDPBB + coculture group.

**Figure 8:**
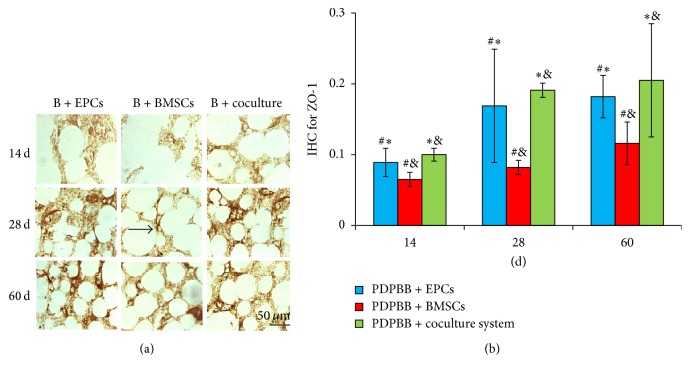
IHC detection and quantitive analysis of ZO-1 in PDPBB groups after implantation. (a) ZO-1 staining in PDPBB seeded with EPCs, BMSCs, and cocultured groups, respectively; B + EPCs, PDPBB + EPCs; B + BMSCs, PDPBB + BMSCs; B + coculture, PDPBB + cocultured system. (b) Quantitive analysis of ZO-1 staining in three groups after implantation. Arrow showed the representative IHC files of ZO-1. Values plotted are means ± SD (*n* = 3). ^*∗*^ *P* < 0.05, versus PDPBB + BMSCs group; ^&^ *P* < 0.05, versus PDPBB + EPCs group; ^#^ *P* < 0.05, versus PDPBB + coculture group.

At 14 dpo, IHC positive OD for CD34 in three groups was gradually increasing from 14 to 28 to 60 dpo. Compared to BMSCs or EPCs group, the OD level of CD34 was upregulated significantly in cocultured group at 14, 28, and 60 dpo, respectively (*P* < 0.05). There was higher OD of CD34 in EPCs group than BMSCs group from 14 to 60 dpo (*P* < 0.05, Figure 6). A similar tendency was observed in detection of OD level for CD105 and ZO-1 IHC (Figures 7 and 8, resp.).

## 4. Discussion

The present data demonstrated that coculture system combined with BMSCs and EPCs had significant advantages of (i) upregulating the mRNA expression of VEGF, Osteonectin, Osteopontin, and Collagen Type I and (ii) increasing ALP and OC amount compared to either of the BMSCs or EPCs only group* in vitro*. Moreover, IHC staining for CD105, CD34, and ZO-1 increased significantly in the implanted PDPBB seeded with coculture system, BMSCs, and EPCs, compared to that seeded with BMSCs or EPCs only, following transplantation. The above results revealed that combination of PDPBB with BMSCs and EPCs promoted osteoblast and angiogenesis* in vitro* and* in vivo*.

Evidences reveal that VEGF, as a key angiogenic and paracrine angiogenic factor, has the ability to (i) simultaneously promote osteogenesis and angiogenesis [27, 28]; (ii) specifically target vascular endothelial cells and subsequently induced endothelial angiogenesis [29, 30]; (iii) increase the permeability of small veins and venules [31]. It is well known that ALP, Collagen Type I, and Osteonectin are the major biological markers in osteoblasts differentiation. Our study showed that ALP activity increased significantly by the coculture of BMSCs and EPCs compared to BMSCs or EPCs alone. Moreover, the staining of Collagen Type I and Osteonectin was remarkably upregulated by the combination of BMSCs and EPCs compared to that of BMSCs or EPCs alone. Given those, the present results showed that there was a positive effect of cocultured system on the mRNA and protein expression of ALP, OC, VEGF, Collagen Type I, Osteopontin, and Osteonectin, which suggested that combination of BMSCs and EPCs would induce osteoblast proliferation, enhancing angiogenic factor expression better compared to BMSCs or EPCs alone. In cocultured system, EPCs enhanced the osteogenesis ability of BMSCs significantly.

The present data also demonstrated that IHC positive staining for CD105, CD34, and ZO-1 significantly increased in the implanted PDPBB seeded with coculture BMSCs and EPCs, compared to that with BMSCs or EPCs only group, respectively. Furthermore, OD values of the selected endothelial cell (EC) and angiogenesis markers, CD105, CD34, and ZO-1, increased from 14 to 28 to 60 dpo in coculture system. Previous study showed that in normal human tissues CD34 was EC marker in various vascular beds [32], while ZO-1 modulated cellular migration and angiogenesis via paracrine regulation by miR-191 [33]. Reports revealed that CD105, a hypoxia-inducible protein associated with proliferation abundantly located in angiogenic endothelial cells, was a promising vascular target used for angiogenic diseases [34].

Abundant studies have demonstrated that EPCs, which can differentiate into endothelial cells, play an indispensable role in neovascularization and vascular maintenance and repair. Factors generated by endothelial cells are also considered crucial for the process of osteogenesis [35]. Previous data also demonstrated that coimplanted EPCs with MSCs increased neovascularization and the capillary score as compared to the MSC only group, which enhanced regeneration of tissue-engineered bone [35]. Reasonably, the present data demonstrated EPCs and BMSCs mutually promoted ability in neovascularization in the cocultured system and endowed PDPBB with the angiogenesis ability by releasing chemotactic factors [36].

It is demonstrated that combination of three key regenerative factors, including a scaffold, reparative cells, and growth factors, is crucial for efficient restoring of bone defects. Nowadays, a number of biomaterials have been innovated, improved, and applied clinically as promising scaffolds for engineering bone [37, 38]. As typical reparative cells, EPCs and BMSCs have been recently determined as potential elements applied to regeneration and repair of tissue after defection [37]. Promising advanced scaffold systems utilised in engineering bone should enable bone defect repairing with fewer scars, decreased morbidity and reject rate, and less pain and less disruption of the soft tissue envelope. For this reason, the engineering scaffolds should be developed to be moldable, biodegradable, and injectable [37]. PDPBB, designed in this research and seeded with BMSCs and EPCs, appeared more biodegradable, moldable, and injectable than with BMSCs or/and EPCs alone. Further SEM micrograph demonstrated that, in contrast to EPCs or BMSCs alone seeded in PDPBB, cocultured cells in PDPBB were well attached and spread throughout the scaffold. These results indicated that the PDPBB with cocultured system possessed favorable cytocompatibility and provided suitable circumstances for cell growth.

## 5. Conclusions

In conclusion, the present study provided evidence that the combination of PDPBB with BMSCs and EPCs promoted osteoblast and angiogenesis. The implanted PDPBB seeded with BMSCs and EPCs might accelerate bone healing by promoting vascularized biological bone regeneration. According to the presented data, we propose that this scaffold system has the potential to provide reconstruction for patients suffering from bone defection. Our findings showed that coculturing BMSCs and peripheral blood derived EPCs may prove useful for generating osteogenic and vascularized bone tissue for clinical use.

## Supplementary Material

Supplementary Material described the cell proliferation of the co-cultured cells before (*in vitro*) and after seeding on PDPBB (*in vivo*). The data revealed that co-cultured group combined with EPCs: BMSCs at ratios of 1:2 achieved the most excellent proliferation activity among the groups. After seeding, the co-cultured group achieved the most excellent proliferation activity among the groups and showed significantly different compared to others.

## Figures and Tables

**Table 1:** Primer and PCR conditions used for qRT-PCR.

Genes	Primer sequence	Lengths of products
VEGF	Forward: 5′ GAAGAAGGAGACAATAAACCC 3′ Reversal: 5′ ACCAGAGGCACGCAGGAA 3′	152 bp

Osteopontin	Forward: 5′ GCTCAGCACCTGAATGTACC 3′ Reversal: 5′ CTTCGGCTCGATGGCTAGC 3′	247 bp

Osteonectin	Forward: 5′ CTCCAGCTGGACTACATCG 3′ Reversal: 5′ CTCCATGGGGATGAGTGGT 3′	369 bp

Collagen Type I	Forward: 5′ TCAACGGTGCTCCTGGTGAAG 3′ Reversal: 5′ GGACCTTGGCTACCCTGAGAA 3′	514 bp

*β*-actin	Forward: 5′ GCTCGTCGTCGACAACGGCTC 3′ Reversal: 5′ CAAACATGATCTGGGTCATCTTCTC 3′	353 bp

**Table 2:** Preparation of partially deproteinized bone.

Reagent/treatment	Processing time	Temperature
30% H_2_O_2_	72 h^*∗*^	RT
Distilled water	30 min	RT
Ethanol	24 h	RT
Distilled water	30 min	RT
Acetone	24 h	RT
Distilled water	30 min	RT
Dying oven	8 h	RT

^**∗**^30% H_2_O_2_ is changed every 24 h during 72 h.

RT: room temperature.

## Abbreviations

BMSCs: Bone marrow stromal cells
EPCs: Endothelial progenitor cells
PDPBB: Partially deproteinized biologic bone
IHC: Immunohistochemistry
IF: Immunofluorescence
qRT-PCR: Quantitive real-time polymerase chain reaction
ALP: Alkaline phosphatase
OC: Osteocalcin
VEGF: Vascular endothelial cell growth factor
CD34: Hematopoietic progenitor antigen
CD133: PROM1
vWF: von Willebrand Factor
CD29: Integrin beta-1
CD90: Thy-1
OD: Optical density
SEM: Scanning electron microscope.
