# Supplementary material for: The Possible Roles of Biological Bone Constructed with Peripheral Blood Derived EPCs and BMSCs in Osteogenesis and Angiogenesis
Source: Biomed Res Int. 2016 Apr 18;2016:8168943. doi: 10.1155/2016/8168943 (PMC4852345; doi:10.1155/2016/8168943)
Supplement: Supplementary file 1 — Supplementary Material described the cell proliferation of the co-cultured cells before (in vitro) and after seeding on PDPBB (in vivo). The data revealed that co-cultured group combined with EPCs: BMSCs at ratios of 1:2 achieved the most excellent proliferation activity among the groups. After seeding, the co-cultured group achieved the most excellent proliferation activity among the groups and showed significantly different compared to others. [file 8168943.f1.docx]

**Supplemental data**

**Cell proliferation of the co-cultured cells**

After 1, 3, 7, 10, and 14 days of cell culture, cells were collected for viability and proliferation by Wst-1 activity assay. The OD was read at 450 nm using an ELISA plate reader (Bio-Rad) (Yang S, 2003; He xiaoning, 2013). The best ratio of EPCs and BMSCs in co-cultured system was determined by Wst-1 assay. According to the O.D. values proceeded by quantitive analyzed, the growth curve of each group was drawn (Supplemental data, Fig.S1). The O.D. value of each group increased from 1 to 14 days. Co-cultured group combined with EPCs: BMSCs at ratios of 1:2 achieved the most excellent proliferation activity among the groups and showed significantly different compared to others (Supplemental data, Fig.S1).

**Cell proliferation after seeding on PDPBB**

The proliferation of EPCs, BMSCs or co-cultured system after seeding on PDPBB was determined by Wst-1 assay. After 2, 4, 6, 8, 10, 12 and 14 days of cell culture on the surface of PDPBB, cells were collected for viability and proliferation by Wst-1 activity assay. The OD was read at 450 nm using an ELISA plate reader (Bio-Rad) (Yang S, 2003; He xiaoning, 2013). According to the O.D. values proceeded by quantitive analyzed, the growth curve of each group was drawn (Supplemental data, Fig.S2). The O.D. value of each group increased from 2 to 14 days. Co-culture seeded group achieved the most excellent proliferation activity among the groups and showed significantly different compared to others (Supplemental data, Fig.S2).


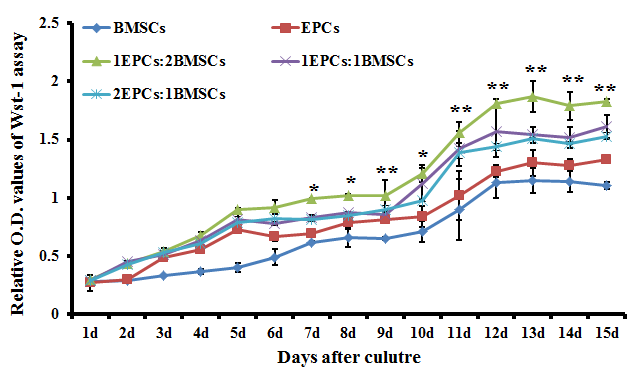


**Fig.S1 Cell proliferation of the co-cultured EPCs and BMSCs**

Values plotted are means ± SD (n=6)

*, vs BMSCs, EPCs, 1EPCs:1BMSCs or 2EPCs:1BMSCs group, *P*<0.05;

**, vs BMSCs, EPCs, 1EPCs:1BMSCs or 2EPCs:1BMSCs group, *P*<0.01

**
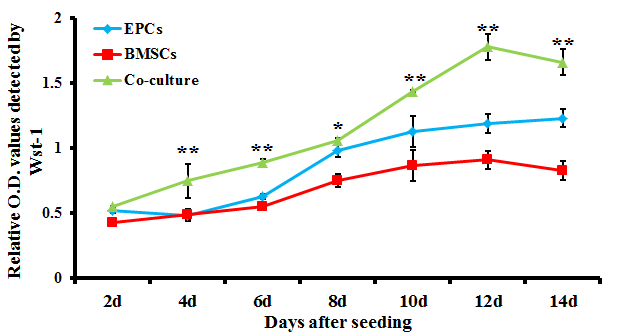
**

**Fig.S2 Activity and proliferation of cells seeded on PDPBB**

Values plotted are means ± SD (n=6)

*, vs BMSCs or EPCs alone group, *P*<0.05

**, vs BMSCs or EPCs alone group, *P*<0.01


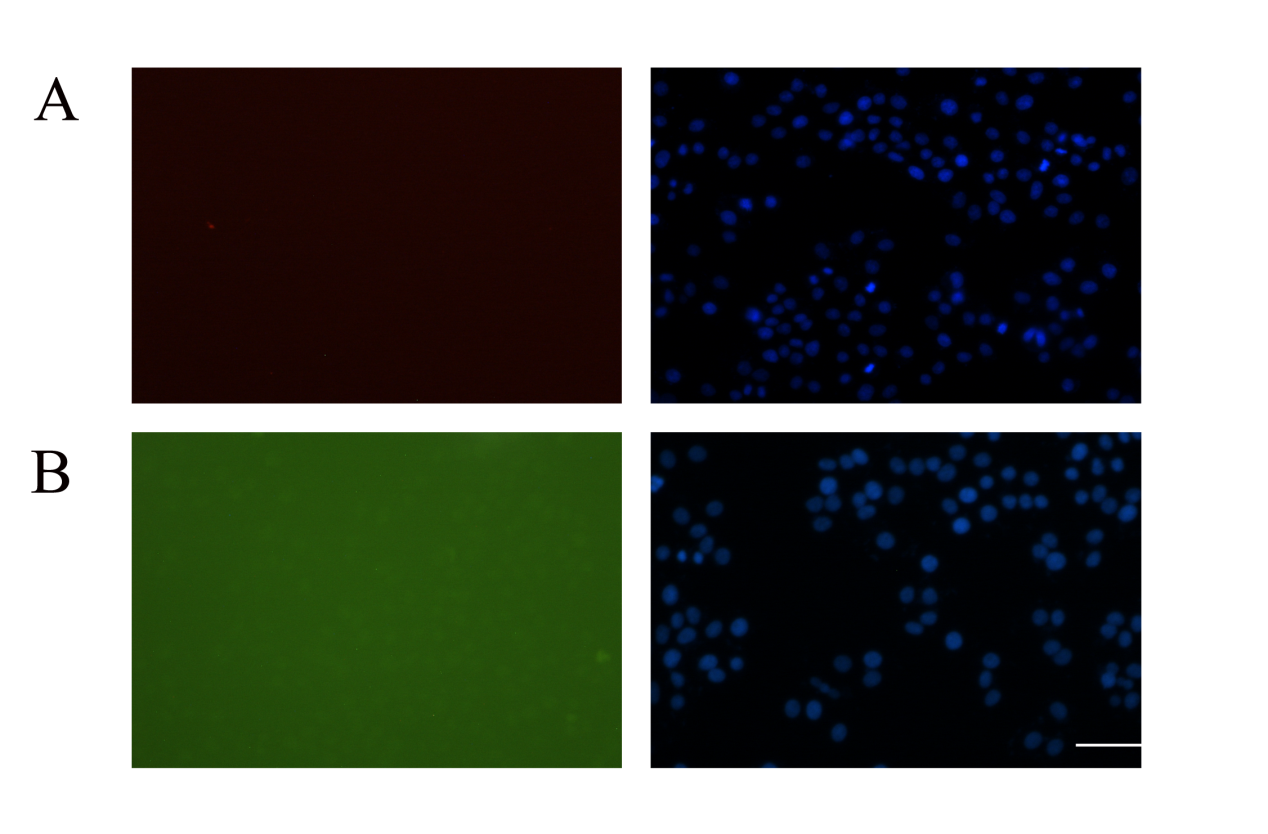


**Fig.S3 Negative control for IF staining of EPCs/BMSCs cell surface markers**

**A,** negative staining of IF for EPCs cell surface markers (red); nucleus were stained by DAPI (blue); **B**, negative staining of IF for BMSCs cell surface markers (green), nucleus were stained by DAPI (blue).

Scale bar: 50μm


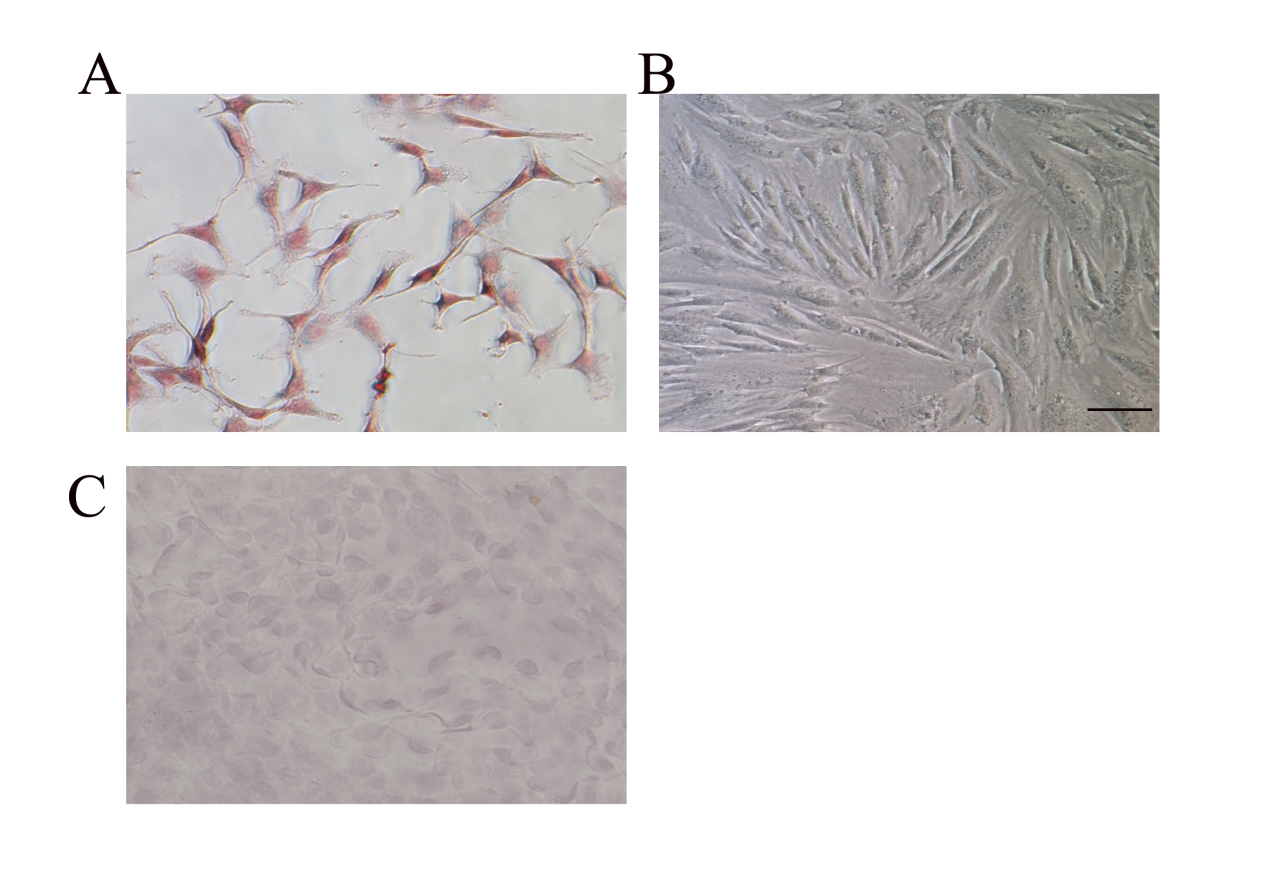


**Fig.S4 Negative control for ALP analysis in different groups**

Negative control ALP staining in EPCs (A), BMSCs (B) and co-cultured groups (C); Scale bar: 50μm
